# Endoscopic surgery of nasopharyngeal angiofibroma

**DOI:** 10.1016/S1808-8694(15)30993-9

**Published:** 2015-10-19

**Authors:** Lidiane Maria de Brito Macedo Ferreira, Érika Ferreira Gomes, Jorge Ferreira Azevedo, João Renato F. Souza, Roberta de Paula Araújo, Adson Sales do Nascimento Rios

**Affiliations:** a MD. ENT Resident at the Fortaleza General Hospital; b Otorhinolaryngologist; Preceptor at the Otorhinolaryngology Residency program of the Fortaleza General Hospital- SESA/SUS; c Oncologist Surgeon, Head of the Head and Neck Surgery Residency - Fortaleza General Hospital - SESA/SUS; d Neuro-endovascular Surgeon; Neurosurgeon - Fortaleza General Hospital - SESA/SUS; e Otorhinolaryngologist; f MD. ENT Resident - Fortaleza General Hospital - SESA/SUS. Fortaleza General Hospital - SESA/SUS

**Keywords:** endoscopic approach, nasopharyngeal angiofibroma

## Abstract

Nasopharyngeal angiofibroma is a vascular benign tumor that affects young men, and surgery is the treatment of choice. Endoscopic surgery has been used to excise tumors in their initial stages, when there is no evidence of residual or recurrent disease.

**Aim:**

The aim of this study is to evaluate the endoscopic approach preceded by tumor embolization as treatment option for stages II to III angiofibroma. Treatment morbidity was evaluated through: surgery duration, hospital stay after surgery, the need for blood transfusion, complications, the time span between preoperative embolization and surgery, and tumor recurrence.

**Methods:**

A prospective study was carried out with nine patients treated at the Fortaleza General Hospital SESA/SUS from October 2001 through November 2004.

**Conclusion:**

Based on the results, we may conclud that the endoscopic approach, when preceded by embolization, is effective to treat angiofibromas in their initial stages, with reduced postoperative morbidity.

## INTRODUCTION

Nasal angiofibroma is a vascular tumor that, despite being histologically benign, it is locally invasive and bears a high rate of persistence and recurrence. It corresponds to 0.5% of head and neck tumors^1^. Its classical treatment is surgery; however there are cases in which we may indicate radiotherapy^2^ or even, hormone therapy.

There are descriptions of atypical locations for this tumor, in sites such as the lacrimal sac, paranasal region, and limited to the sphenoid sinus^3^. Notwithstanding, the most common location is the one limited to he rhinopharynx and nasal cavities.

Its clinical staging is done through the Radkowski, Fisch or Chandler^4^ classification system (Chart - 1).

**Chart 1:**
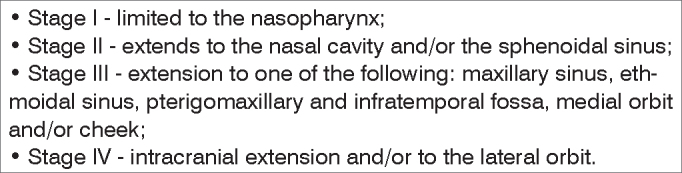
Staging according to Chandler (1984).

Its surgical treatment may be carried out endoscopically or through an open procedure, the latter is through the transpalatine, transmaxillary (involving the mid-facial degloving approach, Weber-Fergusson procedure and lateral rhinotomy), and cranio-facial technique, when there is extensive intracranial tumor invasion. Currently it is advisable to perform a selective tumoral embolization prior to proceeding with any of the techniques known - in order to facilitate surgical access.

Studies have shown that the endoscopic procedure is effective only for the total removal of small and intermediary size tumors^5^, ^6^, ^7^, ^8^; notwithstanding, there are studies that favor even the removal of tumors that extend to the pterigoid fossa and limited to the cranial fossa^9^. However, the debate remains, because studies have shown recurrence of tumors to the infra-temporal fossa or the cavernous sinuses^10^.

The endoscopic approach is, indubitably a sound alternative, because despite being less aggressive to the patient, it causes less morbidity^11^, minimum bleeding, less operative time and greater efficacy^7^, ^12^, moreover, it brings about less transoperative complications and better postoperative function^13^. When properly indicated, the procedure bears low recurrence rates^2^, ^9^, being the first treatment option.

Aiming at assessing the endoscopic approach for the treatment of nasal angiofibromas in its initial stages, in regards to operative morbidity, residual disease and recurrence, we studied patients who were surgically treated because of this tumor at the Fortaleza General Hospital - SESA/SUS.

## MATERIAS AND METHODS

In order to assess the results obtained from the nasal endoscopic resection of nasal angiofibromas in patients who underwent prior tumor embolization, we carried out a prospective analysis of 9 patients who were operated at the Fortaleza General Hospital - SESA/SUS, from October of 2001 to November of 2004, by the same surgeon, under general anesthesia and by the same surgical approach. Only one patient underwent a combined procedure by nasal endoscopy and video-assisted sublabial transmaxillary procedure (patient number seven). All patients were in surgical classes II and III (according to Chandler's classification^4^) (Chart 1).

**Figure 1:**
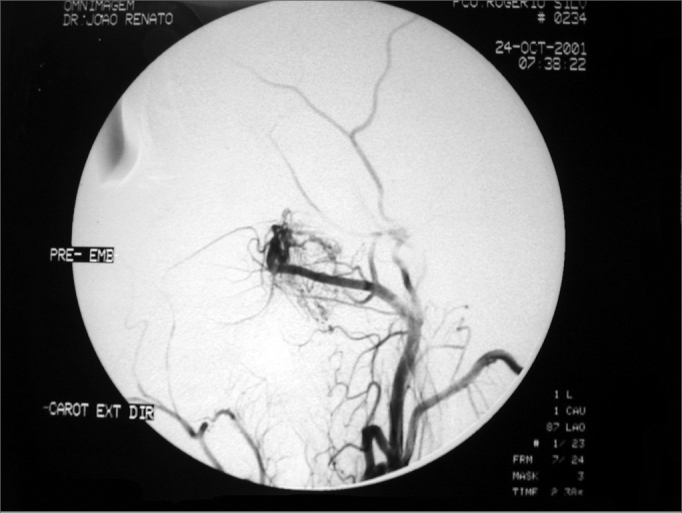
Pre-embolization angiofibroma arteriography - maxillary artery branching off to the tumor.

**Figure 2:**
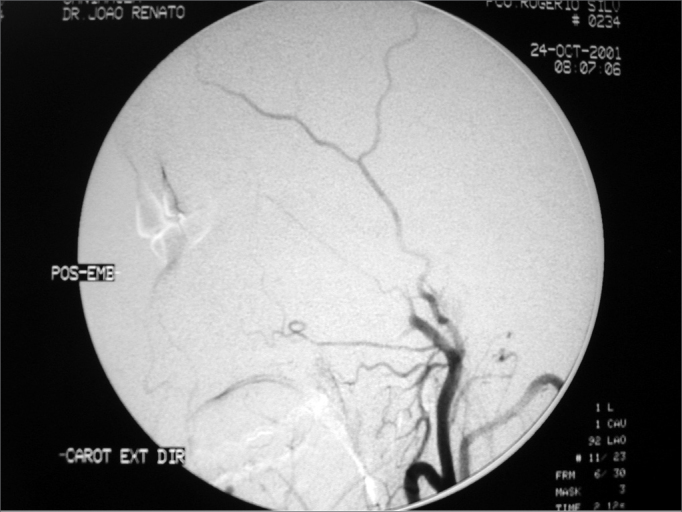
Post-embolization angiography - maxillary artery without tumor branches.

The patients from the present study were between 9 and 20 years of age, with a medical history of epistaxis and nasal obstruction for about 9.5 months in average (1 to 36 months). The most prevalent tumor stage was Stage II (8 patients), however 1 patient was in stage III (Table 1). They all underwent embolization prior to surgery, having an average of 4.7 days between embolization and surgery (from 2 to 7 days) (Figures 1 and 2). Balanced general anesthesia was the type used in all the cases. The surgical procedures were assessed as to the operative time, need for blood transfusion either during or after the procedure, complications, hospital stay after surgery, the time span between embolization and the surgical procedure, and tumor recurrence. The latter was assessed through CT-Scan and/or flexible nasal fibroscopy in the postoperative follow up (which varied from one and thirty four months). The intraoperative bleeding parameters were the volume of liquid in the vacuum container (considering the volume of saline solution used during surgery) as well as clinical parameters such as reflex tachycardia or hypo perfusion. Blood parameters were assessed both in the pre and post operative, and for this study we computed only the levels of hemoglobin. Prior to embolization, all patients were submitted to arteriography. Embolization was carried out through a femoral puncture and introduction of a 6F catheter using Seldinger's technique (a 6F catheter guided by a 035 hydrophilic guide) for a super-selective catheterization of the artery involved (external carotid artery); and with a Renegade® 2.3F micro catheter aided by a 014 Terumo® guide for a super-selective cauterization of the tumor nurturing vessels. Embolization was effective by using 150µ to 250µ and from 250µ to 350µ PVA particles (polyvinyl-alcohol). Embolization complications seen were from the simplest ones such as headaches, trismus, localized pain, fever, rhinorrhea, all the way up to major complications. All patients had their diagnosis confirmed by pathology.

**Table 1:** Relationship between patients, clinical stage, age and disease evolution time.

Pac	Stage	Age	Time of evolution
1	II - Nasopharynx and right nasal cavity.	09 years	02 months
2	II - Nasopharynx with closure of the Rosemüller fossa.	16 years	06 months
3	II - Nasopharynx, right nasal cavity and sphenoid sinus.	20 years	36 months
4	II - Left nasal cavity, sphenoid sinus and nasopharynx.	15 years	12 months
5	II - Left Pterigopalatine Fossa, extending to the cavum and ipsilateral nasal cavities.	15 years	11 months
6	II - Left nasal cavity, extending posteriorly to the choanas, totally occluding them, without cleavage of nasopharynx soft tissue.	15 years	06 months
7	III - Nasopharynx, right nasal cavity, right maxillary sinus, sphenoid sinus, ethmoid cells and medial orbit.	11 years	05 months
8	II - Right Nasopharynx and ethmoid sinus bilaterally.	18 years	07 months
9	II - Right Nasopharynx and right sphenoid sinus.	18 years	01 mês

## RESULTS

**Figure 3:**
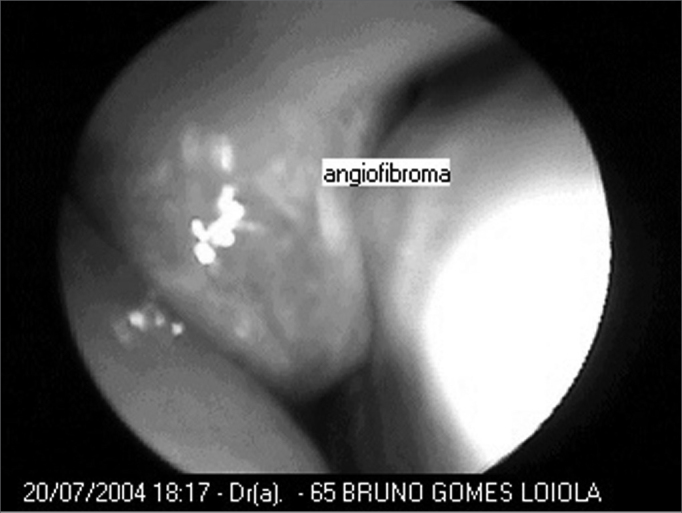
Nasofibroscopy showing the tumor in the right nasal cavity - angiofibroma in the center.

**Figure 4:**
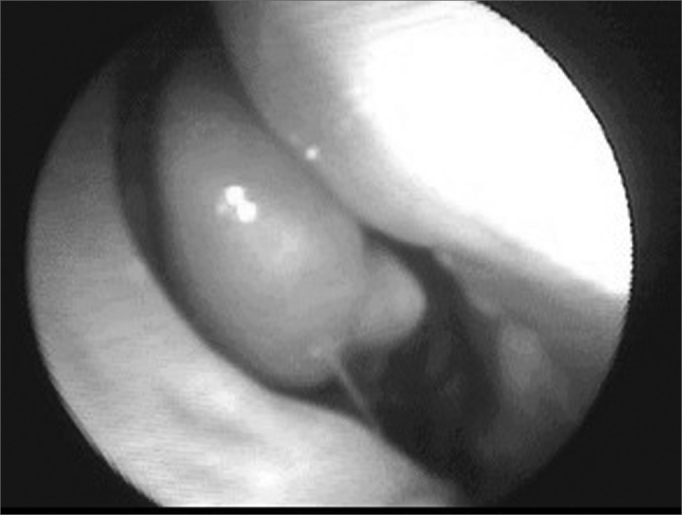
Post-operative nasofibroscopy - no tumor in the right nasal cavity.

Embolization complications were clear in 5 patients (TMJ pain was the most frequently found complication). Average surgery time was of 102 minutes (between thirty minutes and two hours and forty-five minutes) (Table 2). Only one patient required intraoperative blood transfusion, and this same patient was the only one who had a dropping in his hemoglobin levels when pre-op values were compared to postoperative ones (it went from 16 to 14mg/dl, even after transfusion). The other patients kept hemoglobin levels approximately stable (variation of approximately only 1mg/dl). Eight days was the average hospital stay in the postoperative time (between one and seventeen days). Only 3 patients required nasal packing in the postop. There were no surgical complications. Follow up was carried out through CT-Scan or flexible nasal fibroscopy (Figures 3 and 4) for a period of one to thirty-seven months, without evidence of residual disease or recurrence in none of the cases (Table 3).

**Table 2:** Relationship between patients, time span between embolization and surgery, complications and blood transfusion needs and surgery duration.

Patient	Embolization and surgery time	Embolization complications	Surgery	Trans or postoperative transfusion
1	04 days	Vomit and TMJ pain	00:30 h	No
2	06 days	TMJ pain	01:35 h	No
3	03 days	Absent	02:30 h	No
4	04 days	Headaches	02:30 h	No
5	06 days	TMJ pain	01:15 h	No
6	07 days	Absent	01:45 h	No
7	07 days	Absent	02:45 h	Yes (300ml)
8	02 days	Absent	01:00 h	No
9	04 days	Trismus and right temporal headache	01:30 h	No

**Table 3:** Relationship between patients and duration of nasal packing, post-operative hospital stay, tumor recurrence and follow up time.

Patient	Nasal packing	Postoperative hospital stay	Tumor recurrence	Follow up time
1	No	06 days	Absent - CT and nasal fibroscopy	12 months
2	No	13 days	Absent - CT and nasal fibroscopy	18 months
3	02 days	07 days	Absent - nasal fibroscopy	26 months
4	No	06 days	Absent - nasal fibroscopy	19 months
5	No	10 days	Absent - CT and nasal fibroscopy	18 months
6	04 days	11 days	Absent - nasal fibroscopy	09 months
7	04 days	17 days	Absent - nasal fibroscopy	10 months
8	No	01 dia	Absent - nasal fibroscopy	37 months
9	No	02 days	Absent - nasal fibroscopy	03 months

## DISCUSSION

Nasal angiofibroma is a fairly common tumor in young people, thus presenting peculiar characteristics as to its therapeutic aspects. Its treatment is almost exclusively surgical, and all the therapeutic weaponry should be used in order to promote patients’ fast recovery, sparing them as much as possible from school or work absence, thus reducing the social repercussions of the disease. Our series corroborated such behavior as to age of onset, and all the patients were young. Endoscopic nasal surgery is not only feasible, but also a very efficient alternative in the treatment of these tumors it their initial stages, when it is less aggressive and dos not impair much the patients’ postop recovery. Although it has been well established that one hour more of surgery time doubles the incidence of infection and it is certainly one more factor that increases trauma and its repercussions, operative time is still discussed. There is no relation between surgery duration and postoperative complications, death or long term survival^14^. Notwithstanding, it is also known that the longer the surgery takes, greater are the alterations that happen in body homeostasis during surgical trauma. Thus the need to improve techniques in order to spend less time in surgery and reduce the risk to the patient's life. Endoscopic surgery was developed in order to provide a less invasive approach and thus cause less harm to patients. Since this is a nasal approach, endoscopy brought about not only less invasion, but also better cosmetic results and less surgery time. This was seen in the present study, in which average surgical time was of 102 minutes, matching reports considered satisfactory in the literature^7^.

The short hospital stay after surgery also contributes to patients’ fast recovery, reflecting the lack of complications and readmissions^15^. Endoscopic surgery has a great advantage because it requires less rehabilitation days after surgery, requiring less days of hospitalization and is less subject to hospital infections.

However, since it is a very specific technique, nasal endoscopic surgery has an important disadvantage - it requires a bloodless operating field - because any amount of blood may prevent a good visualization and thus preclude the surgical approach.

Tumor embolization is a technique that contributes much to nasal angiofibroma endoscopic surgery, having a critical participation in its vascular composition and making the surgery more accessible and visible for the surgeon. Endoscopic surgery has a very important advantage, which is the fact that it preserves both the anatomy and physiology of the nose, and the prior tumor embolization contributes to a better identification of structures during surgery, without considerable hemorrhage, thus making the procedure easier. Thus, prior embolization contributes to reduce surgical morbidity. Studies state that the ideal time between tumor embolization and the endoscopic surgical procedure should be around 24 to 72 hours^5^; however, in our department this time has varied within an average of 4.7 days, with effective embolization of all the tumors and performance of all the endoscopic procedures without problems. The clinical observation in our clinic, considering the tumoral embolization of over 30 angiofibromas operated, not only through endoscopy, but also through open surgery, it has been between 5 and 7 days, after embolization as the ideal period for surgery, having seen that the tumor becomes more fibrous, with areas of necrosis and with much less bleeding than those operated with less than 3 days of wait, thus making the surgeon's life a lot easier. We also noticed that after 7 days, bleeding returned, this time it was induced by collateral circulation created to nurture the tumor. These findings are in disagreement with the literature, in which most of the authors use resorbable particles such as gelfoam or dextran microspheres or short duration non-absorbable such as Ivalon, ITC contour or Terbal. In our department we use polyvinyl-alcohol particles, which last longer and is more efficient.

Complications and the need for blood transfusion also indicate how severe the surgery should be, as well as the aggression caused to the patient's body. Transoperative transfusion indicates poor prognosis, reducing postoperative survival^14^. The endoscopic surgeries of the present study did not show severe complications in any of the patients, there were only minor complications from the embolization, and only one case which needed blood transfusion. As it happens to all invasive procedures, embolization also bears some risks, which should be duly explained to the patients, and all care should be taken in order to avoid them: the most significant risks are blindness - due to the occlusion of the ophthalmic artery, or due to occlusion of the middle cerebral artery through the collateral circulation.

## CONCLUSION

With the data presented in this study, we may conclude that the endoscopic approach is a safe procedure to resect nasal angiofibromas in its initial stages, having seen its low morbidity and great effectiveness, with total tumor removal without recurrence rates. However, it is worth highlighting that such success is primarily due to tumor embolization prior to surgery, which makes the procedure safe, fast and effective. However, since the number of patients is so small, and recent cases have such a short follow up time, we intend to increase our numbers and the assessment time for a more complete and accurate study.
